# DD-Net: a dual-domain progressive fusion network enhanced by high–low frequency separation for lung nodule segmentation

**DOI:** 10.3389/fmed.2026.1907240

**Published:** 2026-08-13

**Authors:** Xiangsuo Fan, Lihong Deng, Jiachen Hou, Zhougui Ling

**Affiliations:** 1 School of Automation, Guangxi University of Science and Technology, Liuzhou, China; 2 Earthwork Collaborative Innovation Center, Guangxi University of Science and Technology, Liuzhou, China; 3 Department of Medical Imaging, The Fourth Affiliated Hospital of Guangxi Medical University, Liuzhou, Guangxi, China; 4 Department of Medical Imaging, Liuzhou Worker's Hospital, Liuzhou, Guangxi, China

**Keywords:** dual-domain collaboration, frequency features, hybrid loss function, lung cancer, lung nodule segmentation

## Abstract

**Introduction:**

Precise segmentation of pulmonary nodules plays a vital role in differentiating benign from malignant lesions and in tracking their progression over time, however, the substantial variability of nodules in size, morphology, and texture frequently results in either excessive or insufficient segmentation.

**Methods:**

This paper proposes the spatial-frequency dual-domain collaborative segmentation network (DD-Net), which integrates the spatial and frequency domains to enhance the ability to characterize the diverse features of nodules. First, a frequency separation mechanism is introduced during the frequency-domain encoding process to model high- and low-frequency components differentially, balancing the representation of boundary details with overall structural information. Through frequency-domain filtering and learnable weight modulation, it obtains more discriminative frequency representations. Subsequently, an incremental attention fusion process is integrated into the high-level semantic feature interaction stage, enabling gradual alignment and reweighting of spatial and frequency domain features across channels and spatial dimensions. Finally, the traditional feature propagation path is restructured by a frequency self-attention mechanism, which models correlations between different components in the frequency space to help amplify key responses.

**Results:**

The effectiveness of the proposed model was evaluated using the publicly available LIDC-IDRI dataset together with a meticulously annotated private dataset. Experimental results indicate that the proposed approach outperforms multiple state-of-the-art methods on both datasets, achieving IoU/Dice scores of 88.02%/92.51% on the public LIDC-IDRI dataset and 65.03%/77.08% on the highly challenging private dataset.

**Conclusion:**

The proposed DD-Net employs frequency separation and attention fusion mechanisms for accurate lung nodule segmentation, demonstrating great potential for early lung cancer screening.

## 1 Introduction

Lung cancer is among the most prevalent malignant tumors worldwide and remains one of the leading causes in terms of both incidence and mortality (1), resulting in nearly 1.8 million deaths each year (2). Early detection is essential for enhancing patient prognosis and extending survival time. Pulmonary nodules are among the most frequent indicators of early-stage lung cancer. Their identification enables quantitative assessment of nodule size and density, assists in differentiating benign from malignant features, and supports the prediction of growth patterns (3, 4). Accurate delineation of lung nodules forms the cornerstone of these downstream analytical tasks (5). With the ongoing development of medical imaging technologies, a vast amount of imaging data has provided crucial support for lung nodule segmentation research, with CT and MRI having become relatively mature and widely used diagnostic tools (6). However, since lung nodules are typically small in size and exhibit characteristics such as strong textural heterogeneity, blurred boundaries, and structural adhesions (7), constructing models with high-accuracy segmentation capability across diverse nodule types continues to be a difficult problem in medical image processing.

Image segmentation occupies a crucial position in medical image analysis. It intends to partition medical scans into several regions with clear clinical significance. This process acts as an essential prerequisite for numerous medical practice scenarios, such as clinical disease diagnosis and image-assisted surgical treatment (8). Although manual segmentation performed by experienced clinicians is generally considered a reliable method, it still has numerous limitations. For example, insufficient consistency among different annotators or annotation groups can lead to poor result stability, and human errors, omissions, or misjudgments of nodule features may also occur (9). Furthermore, lung nodules exhibit slight morphological and phenotypic variations with rich diversity, which creates great difficulties for manual segmentation, especially when complicated by intricate anatomical conditions including vascular penetration and pleural adhesion. Given these issues, it is extremely essential to build a computer-aided diagnosis (CAD) system for auxiliary lung nodule segmentation tasks.

Current lung nodule segmentation methods primarily rely on architectures based on convolutional neural networks (CNNs). However, convolutional operations are constrained by limited local receptive fields, rendering them incapable of effectively modeling long-range dependencies and prone to over-segmenting non-nodular regions due to misclassification (10). In contrast, the Vision Transformer (ViT), which is based on attention mechanisms, can aggregate feature information on a global scale, helping to improve the ability to distinguish between nodule regions and surrounding tissue. However, such methods are relatively deficient in capturing local details and often perform poorly in terms of boundary refinement (11). Most existing segmentation methods primarily operate in the spatial domain. However, incorporating frequency-domain information has the potential to provide important support for the accurate identification of various fine-grained features.

In relevant research incorporating frequency-domain information, most methods model features across different frequency bands uniformly, lacking explicit differentiation and targeted characterization of high-frequency and low-frequency information. The limitation of this approach is that the boundary and fine-grained texture information carried by high-frequency components is easily weakened, while the overall structure and semantic distribution expressed by low-frequency components are also difficult to fully utilize. Furthermore, frequency-domain features are more abstract than spatial-domain features. Traditional operators based on local receptive fields, such as convolution, have significant shortcomings when modeling multi-frequency interactions, making it difficult to effectively capture cross-scale and global dependency information.

Furthermore, the high diversity of lung nodules in terms of morphology, size, and boundary characteristics poses significant challenges to the development of a unified and robust segmentation model. The model must not only adapt to the structural differences among various nodule types but also maintain precise identification of border regions within complex anatomical contexts. Simultaneously, differences in the expression forms and information distribution across scales and domains further complicate the modeling of feature collaboration, often leading to insufficient representation of critical information. Therefore, how to realize the effective coordination and progressive fusion of multi-scale spatial-frequency dual-domain information remains a critical issue requiring urgent resolution.

Although existing studies have achieved substantial progress in the field of lung nodule segmentation, several critical challenges remain unresolved. First, existing spatial-domain-based algorithms struggle to simultaneously preserve fine-grained boundary details and capture global contextual information. This limitation becomes particularly prominent when dealing with nodules exhibiting irregular morphology, ambiguous boundaries, and complex surrounding tissue environments. Second, current frequency-domain-based methods generally treat frequency information as a unified representation, without explicitly distinguishing and modeling high-frequency and low-frequency components. Consequently, these methods fail to simultaneously enhance boundary details and maintain the structural integrity of lesions. Third, existing approaches typically employ simple attention mechanisms to achieve interactions between spatial-domain and frequency-domain features. Such fusion strategies may introduce redundant information and weaken the representation of critical features. Moreover, current frequency-domain modeling schemes are insufficient in capturing long-range dependencies among different frequency components, resulting in ineffective utilization of global frequency information.

To address the aforementioned challenges, we propose a high–low frequency separation-enhanced dual-domain progressive fusion network (DD-Net) for lung nodule segmentation, aiming to achieve more accurate and robust segmentation performance. The main contributions of this study are summarized as follows:

(1) We propose a spatial-frequency dual-domain collaborative modeling framework that introduces a high-low frequency separation mechanism (FreqSep) in the frequency-domain encoder to perform differentiated modeling of diverse frequency components. Compared to a unified frequency-domain representation, this method can simultaneously account for both boundary details and overall structural information, thereby enhancing adaptability to diverse nodule morphologies.

(2) We design a Progressive Attention Fusion (PAF) module that achieves gradual fusion of spatial and frequency domain features through staged attention modulation, highlighting responses in key regions and reducing interference from irrelevant information during the fusion process.

(3) We designed a Frequency Self-Attention (FSA) module to model the relationships between different frequency components in the frequency domain and to enhance important features, which helps better preserve nodule fine-grained structural information during reconstruction.

(4) Extensive experiments conducted on both public and private datasets demonstrate that the proposed DD-Net achieves a favorable balance between segmentation accuracy and computational efficiency, providing reliable support for early lung cancer screening and computer-aided diagnosis.

## 2 Related work

### 2.1 Lung nodule segmentation

Lung nodule segmentation essentially falls within the field of image segmentation. Currently, mainstream approaches fall into two major categories: traditional processing methods and deep learning methods. Traditional approaches are primarily dependent on hand-designed features and rules, achieving segmentation by analyzing low-level image information such as grayscale and texture. Common techniques include threshold segmentation (12), region growing (13), edge detection (14), active contour models (15), and watershed transformation (16). However, these methods struggle to extract visually discriminative representations to address the challenges of the task and have limited generalization capabilities.

In contrast, deep learning-based segmentation methods, which replace traditional machine learning approaches with CNNs, are better suited for a variety of lung nodule segmentation tasks. Tang et al. (17) proposed Convunet for lung nodule segmentation. By introducing DSC-Blocks based on deep separable convolutions to replace traditional attention mechanisms and modeling feature interactions via Hadamard products, the method enhances the model's feature representation capabilities while reducing computational complexity. This approach attained 88.90% Dice score when evaluated on the LUNA16 dataset. Cai et al. (18) proposed MDFN, a multi-branch architecture for lung nodule segmentation tasks. This model integrates a Multi-Scale Spatial and Channel Feature Selection (MSCFS) module together with a self-calibrated encoder, so as to strengthen the modeling capability for key regions and boundary details in complex CT images. On the LUNA16 dataset, the results reached 80.78% in IoU and 89.19% in Dice coefficient. Li et al. (5) proposed a lightweight medical image segmentation network, FOC-Net, which significantly improved lung nodule segmentation performance while maintaining computational efficiency by introducing spatial displacement, wavelet downsampling, and attention fusion mechanisms. This method attained an IoU score of 85.37% and 92.06% Dice score on the LIDC-IDRI dataset. Abulizi et al. (19) proposed MBM-UNet, which introduces a multi-branch feature extraction module (MBVSS) based on VM-UNet, incorporates the CBAM attention mechanism into the bottleneck layer, and adopts a hybrid loss function that combines binary cross-entropy with Dice loss, effectively improving the accuracy of lung nodule segmentation. It achieved 84.51% IoU and 91.82% Dice coefficient when evaluated on the LIDC-IDRI dataset. However, our DD-Net model achieves dual-domain feature co-modeling through high- and low-frequency separation and progressive attention fusion, achieving 88.02% IoU and 92.51% Dice on the LIDC-IDRI dataset.

### 2.2 CNN-transformer hybrid models

Compared to Convolutional Neural Networks (CNNs), Transformer-based architectures typically entail higher computational costs (20). At the same time, the lung nodule segmentation task itself relies on contextual information regarding the target and its surrounding areas, making it necessary to balance local and global feature modeling. Therefore, modeling long-range dependencies requires a more reasonable balance between expressive power and computational resource consumption. Against this backdrop, hybrid architectures that fuse CNNs with Transformers have become a key research trend. For example, UCTransNet, developed by Wang et al. (21), introduces a channel Transformer to replace traditional skip connections and utilizes channel-crossed attention to fuse multi-scale features, thereby mitigating semantic gaps and improving segmentation performance. TransUnet, proposed by Chen et al. (22), employs self-attention for global modeling and reformulates the segmentation task as a masked classification problem. By introducing learnable queries and combining cross-attention with multi-scale CNN features for layer-by-layer refinement, it improves segmentation accuracy through a coarse-to-fine approach. Similarly, Lan et al. (23) developed BRAU-Net++, which introduces two-layer routed attention (BRA) to model dynamic sparse long-range dependencies and employs a BiFormer module for efficient feature extraction while reducing model complexity. However, these methods still primarily rely on the QKV mechanism, combined with convolutional operations to supplement local information. Nevertheless, for the lung nodule segmentation task, relying solely on global modeling in the spatial domain remains limited. Conversely, incorporating frequency information into the spatial representation process and achieving more refined collaborative modeling between local details and global dependencies is more conducive to fully capturing the target structure.

### 2.3 Utilization of frequency-domain information

Different lesion areas in medical imaging often show distinct frequency distribution characteristics, which serve as important cues for precise segmentation. Specifically, low-frequency components primarily depict the global structure and tissue contours of the image, while high-frequency components focus on edge information and fine-scale variations, reflecting subtle structural features (24). Frequency-domain information can be transformed into discriminative feature representations, providing effective assistance and guidance for image segmentation (25). Farshad et al. (26) proposed Y-Net, which introduces a spectral encoder and utilizes Fast Fourier Convolution (FFC) to model features in the frequency domain, thereby enhancing the ability to capture global contextual information. Li et al. (27) proposed the Global Frequency-domain Network (GFUNet), which constructs a dual-domain encoding framework for effective exploitation of frequency-domain features, consequently enhancing the segmentation model's overall capability. Geng et al. (28) proposed BDFNet, which introduces a Dual-Stream Frequency Attention Module (DFAM) to learn frequency-aware attention weights and incorporates a boundary-aware attention mechanism to explicitly enhance feature representations in lesion boundary regions, thereby improving medical image segmentation performance. Zhou et al. (29) proposed SF-UNet, which decomposes features into high-frequency and low-frequency components through the FSA module and enhances frequency-domain representations using adaptive filtering. Similarly, Kong et al. (30) proposed FFTformer, which introduces the frequency domain-based self-attention solver (FSAS) to transfer Transformer attention computation into the frequency domain for reducing computational complexity, while employing the DFFN to optimize frequency-domain feature representation. Different from these methods, our approach explicitly separates input features through the FreqSep module and adaptively enhances high-frequency information containing boundary details and low-frequency information representing overall anatomical structures. Meanwhile, the FSA module is incorporated to capture long-range dependencies among different frequency components, thereby refining the global frequency-domain representation. This enables collaborative modeling of lesion boundary details, global structural information, and long-range contextual dependencies.

### 2.4 Feature fusion

In medical image analysis, accurately capturing subtle structural changes and key regions is crucial for model performance. Therefore, feature fusion is widely used to integrate representations from different information sources, forming more comprehensive and discriminative representations by effectively combining diverse features (31). Especially in tasks involving the identification of small lesions or key regions, introducing feature fusion mechanisms has become a key approach to improving model performance. For example, Jiang et al. (32) proposed iU-Net, which uses dual encoders to separately extract global semantics from a Transformer and local details from a CNN, and employs a feature fusion module based on wavefunction representations to achieve unified modeling of cross-spatial information. Xie et al. (33) introduced Multi-Scale Dense Convolution (MSDC) modules and Local Attention Modules (LAM) based on U-Net to achieve multi-scale context fusion and key region enhancement. Rezvani et al. (34) proposed FusionLungNet, which effectively enhances boundary representation capabilities by combining Channel Attention (CAA) and a Multi-Scale Fusion Module (MFF) with a residual refinement mechanism. However, a common limitation of these methods is the lack of a gradual guidance mechanism in the fusion process, which can easily introduce redundancy or weaken critical structures. In contrast, our proposed Progressive Attention Fusion (PAF) module employs a phased fusion strategy comprising attention screening, feature compression, and re-attention enhancement. This approach achieves cross-domain progressive fusion of spatial and frequency domain features at the high-level semantic stage, thereby enhancing the effectiveness and robustness of the fused representations.

## 3 Method

### 3.1 Overall architecture

**Figure 1:**
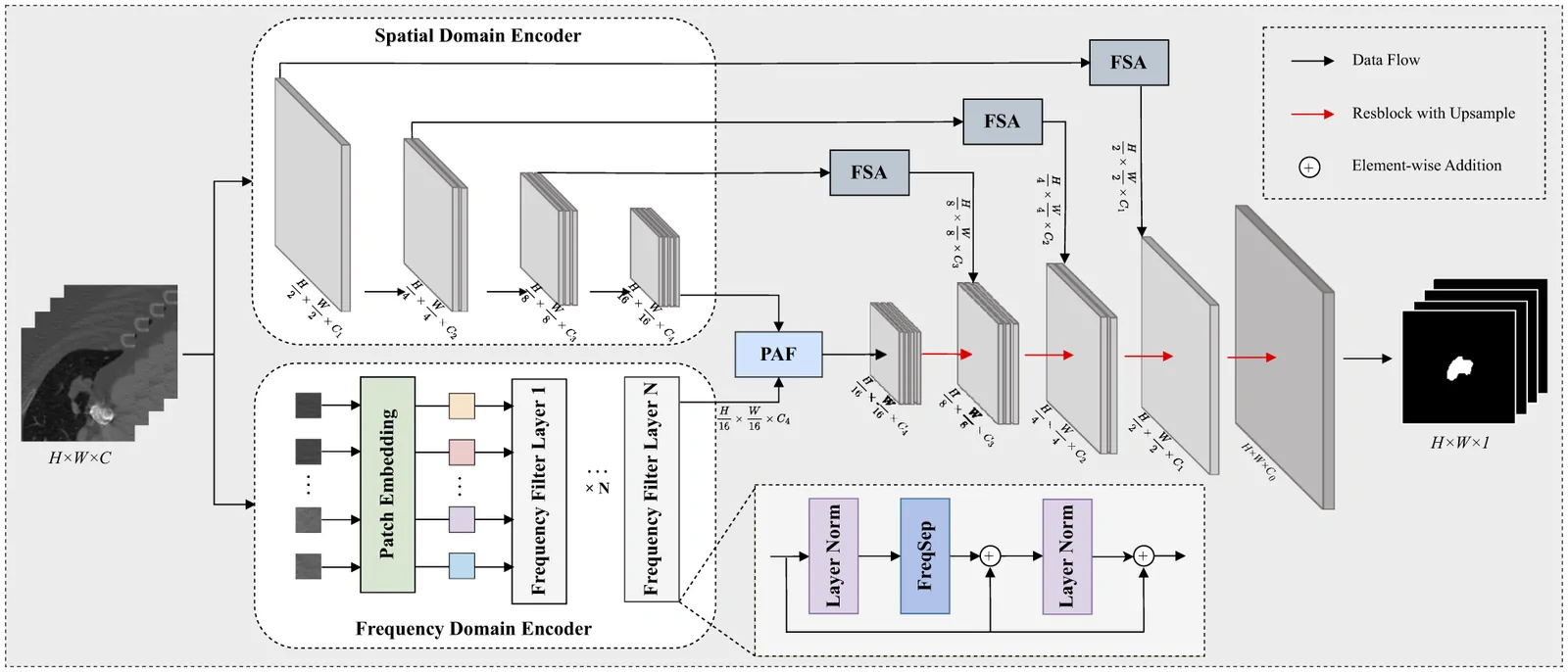
Overall architecture of DD-Net. By jointly modeling spatial and frequency domain features, and combining cross-domain Progressive Attention Fusion (PAF) with Frequency Self-Attention (FSA), the model achieves layer-by-layer enhancement and effective fusion of dual-domain features, thereby significantly improving segmentation accuracy and boundary localization capabilities.

The overall architecture of DD-Net is shown in Figure 1. This network performs collaborative modeling of spatial and frequency domain features, constructing a parallel dual-encoder architecture. The spatial encoder is based on ResNet34 (35), extracting multi-scale structural representations layer by layer. The frequency-domain encoder first performs patch embedding on the input, and then conducts frequency-domain feature modeling through N layers of Frequency Filter Layers (in our experiments, we set N = 12). During this process, a frequency separation module (FreqSep) is integrated to enable differentiated modeling of high- and low-frequency components, enhancing the representation of frequency information and capturing global contextual dependencies. Specifically, given an input image *I*∈ℝ^*H*×*W*×*C*^, where *H*,*W*, and *C* represent the image's height, width, and number of channels, respectively. After channel mapping, the input is simultaneously fed into the spatial encoder and the frequency-domain encoder. The spatial encoder progressively extracts multi-scale spatial domain features through a hierarchical ResNet34 structure, denoted as $E_{i}\in {\mathbb{R}}^{\frac{H}{2^{i}}\times \frac{W}{2^{i}}\times C_{i}}$, where *i*∈{1, 2, 3, 4}. The frequency-domain encoder models the input in the frequency domain to generate a frequency feature representation *F*_*f*_, which is used to capture the global structural information and long-range dependencies of the nodule region, thereby forming a spatial and frequency dual-domain feature representation.

**Figure 2:**
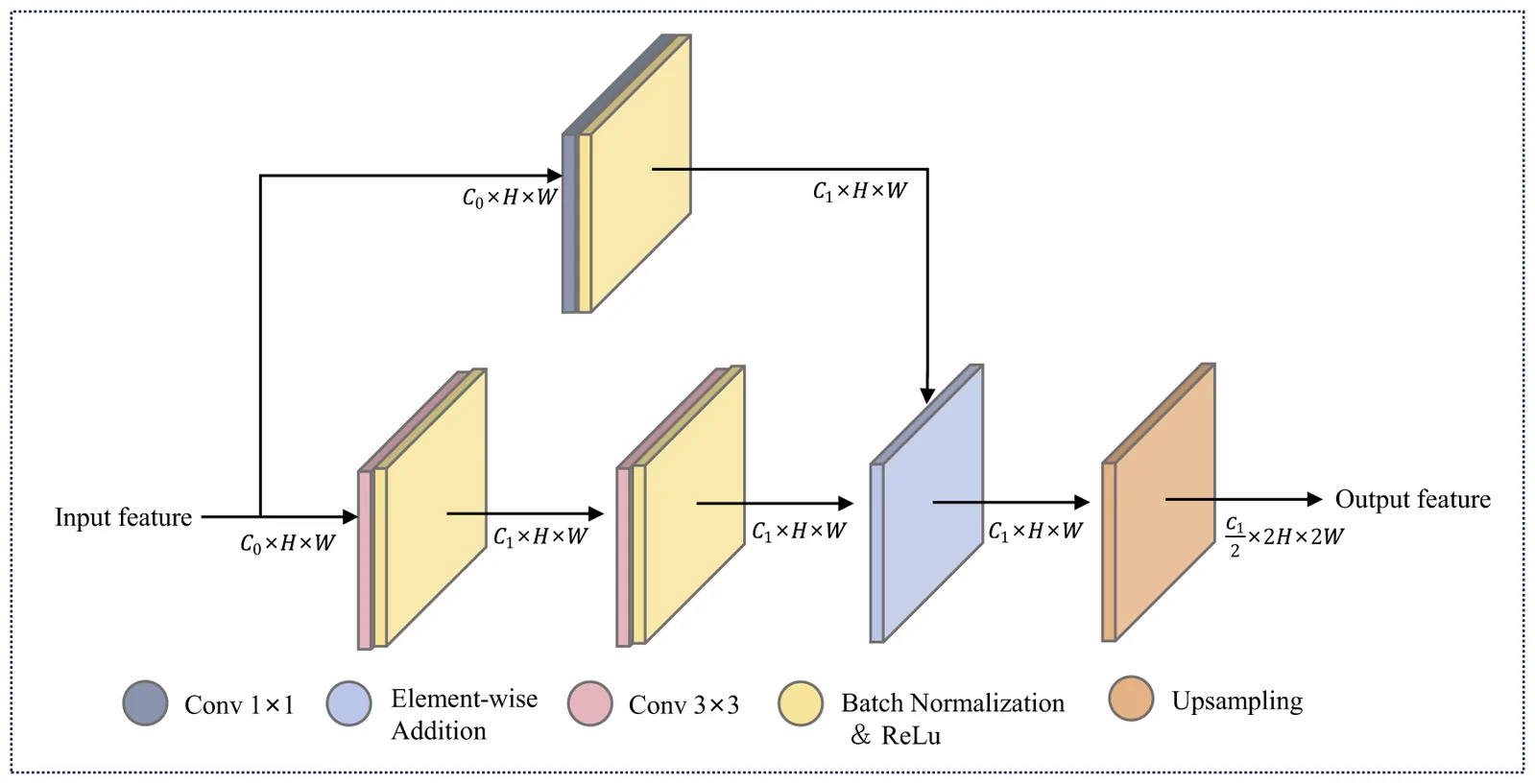
Structure of Resblock with Upsample.

Subsequently, a Progressive Attention Fusion (PAF) module is introduced between the top-level spatial semantic features *E*_4_ and frequency features *F*_*f*_ to achieve progressive fusion of spatial and frequency features. This module selectively enhances the fused features through the collaborative modeling of channel attention and spatial attention, thereby reinforcing the overall structure and key response representations of the nodule regions. Concurrently, a Frequency Self-Attention (FSA) module is embedded in the feature propagation path to model global dependencies among frequency components. During decoding, a Resblock with Upsample (structure shown in Figure 2) is introduced to progressively restore spatial resolution and refine feature representations. Ultimately, through the progressive fusion of multi-level features, the network outputs segmentation results *O*. The FreqSep, PAF, and FSA modules will be described in detail below.

### 3.2 Frequency separation module (FreqSep)

In frequency representation, different frequency bands carry distinct information: high-frequency components primarily correspond to edge and texture details, while low-frequency components characterize overall structure and semantic distribution (24). In the lung nodule segmentation task, nodule boundaries and fine-grained morphology rely on high-frequency information, whereas their overall contours and spatial distribution are more strongly represented by low-frequency components. Following the transformation of spatial-domain features into the frequency domain, the features can be divided according to their frequency properties, enabling more specialized feature representation learning. Frequency filtering mechanisms play a key role in this process. By constraining and modulating predefined frequency bands, they highlight important structural information and suppress redundant responses (36). Specifically, high-pass filtering emphasizes high-frequency regions far from the spectral center to enhance detail representation, whereas low-pass filtering retains low-frequency components near the center region to preserve overall structural coherence (37).

**Figure 3:**
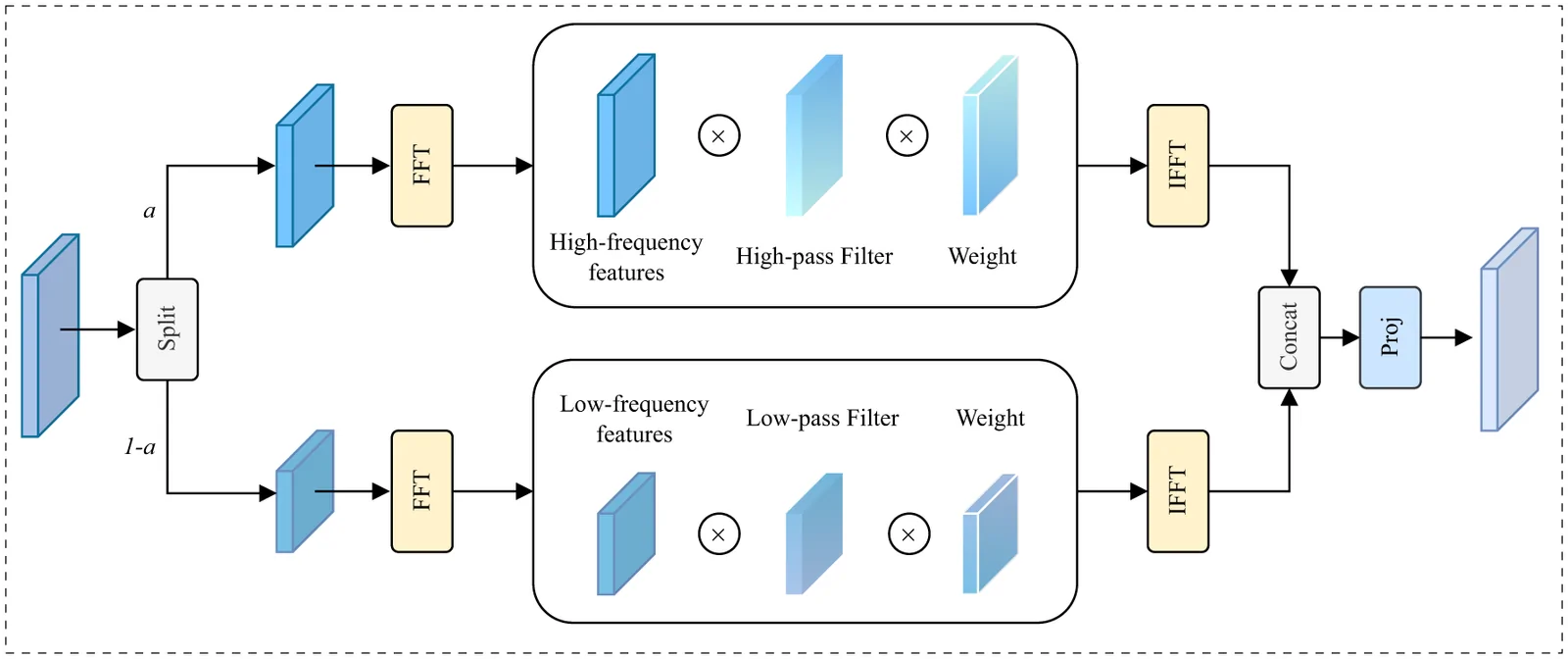
Shows the structure of the FreqSep module, the key component of the Frequency Filter Layer, which aims to separate and enhance high-frequency and low-frequency features within the frequency domain.


As presented in Figure 3, during the frequency-domain encoding process of DD-Net, we designed a frequency separation module (FreqSep) in the Frequency Filter Layer to achieve differentiated modeling of high- and low-frequency information. Combined with learnable frequency weights for modulation, this enables the network to balance the depiction of local details with the expression of global structure, thereby obtaining more discriminative frequency representations. For the input feature *M*∈ℝ^*H*×*W*×*C*^, we first divide it into two parts along the channel dimension according to the ratio *a*∈(0, 1), yielding $M_{1}\in {\mathbb{R}}^{H\times W\times aC}$ and $M_{2}\in {\mathbb{R}}^{H\times W\times \left(1-a\right)C}$. Subsequently, we apply the Fast Fourier Transform (FFT) to each part separately to obtain the corresponding frequency-domain representations *M*_*h*_ and *M*_*l*_. In the frequency domain, we introduce high-pass and low-pass filtering masks to constrain the two branches and modulate them using learnable complex weights. Afterward, the inverse fast Fourier transform (IFFT) is then applied to transform the refined features back into the spatial domain. The above process can be formally expressed as Equations 1–5:

$$\begin{array}{l} M_{h}=\text{FFT}\left(M_{1}\right),\;M_{l}=\text{FFT}\left(M_{2}\right) \end{array}$$ (1)

$$F_{h}\left(x,y\right)=\{\begin{array}{cc} 0, & \sqrt{{\left(x-x_{0}\right)}^{2}+{\left(y-y_{0}\right)}^{2}}<r\\ 1, & \text{otherwise} \end{array}$$ (2)

$$F_{l}\left(x,y\right)=\{\begin{array}{cc} 1, & \sqrt{{\left(x-x_{0}\right)}^{2}+{\left(y-y_{0}\right)}^{2}}<r\\ 0, & \text{otherwise} \end{array}$$ (3)

$$\begin{array}{l} M_{h}^{\prime }=M_{h}⊙F_{h}⊙W_{h},\;M_{l}^{\prime }=M_{l}⊙F_{l}⊙W_{l} \end{array}$$ (4)

$$\begin{array}{l} M_{1}^{\prime }=\text{IFFT}\left(M_{h}^{\prime }\right),\;M_{2}^{\prime }=\text{IFFT}\left(M_{l}^{\prime }\right) \end{array}$$ (5)

In this context, *F*_*h*_ and *F*_*l*_ denote the high-pass and low-pass filtering masks, respectively. (*x*_0_, *y*_0_) denotes the spectral center position, *r* controls the filtering range, and *W*_*h*_ and *W*_*l*_ are the corresponding complex weight parameters. Finally, the outputs of the two branches, $M_{1}^{\prime }$ and $M_{2}^{\prime }$, are concatenated along the channel dimension, and feature integration is achieved through a linear mapping.

### 3.3 Progressive attention fusion module (PAF)

In DD-Net, spatial-domain features and frequency-domain features exhibit significant differences in response patterns and information distribution. Direct superposition can easily introduce redundant responses and weaken the expression of key structural information. To overcome this limitation, we designed the Progressive Attention Fusion (PAF) module as an information interaction unit connecting spatial and frequency domain features, embedded in the higher layers of the encoder. The PAF module integrates cross-domain high-level semantic features and, through a two-stage progressive parallel attention modulation mechanism, gradually guides effective responses and suppresses redundant information during feature interaction.

**Figure 4:**
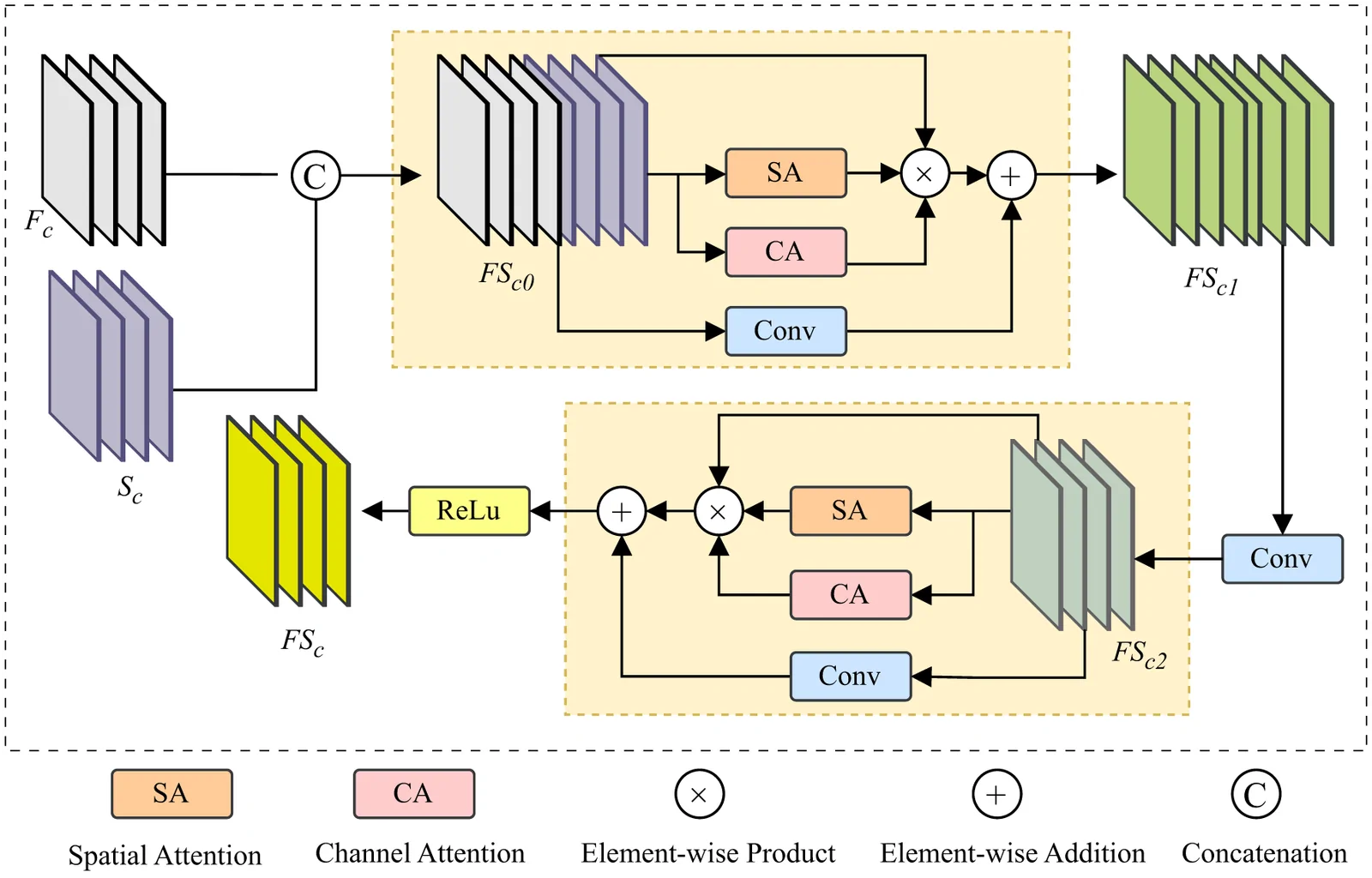
Architecture of the PAF module. Through the synergy of channel and spatial attention, cross-domain features are progressively fused and enhanced.

The architecture of PAF is illustrated in Figure 4, fuses *S*_*c*_ and *F*_*c*_ extracted from spatial- and frequency-domain encoders via channel-wise concatenation to produce the initial fused representation, as shown in Equation 6:

$$\begin{array}{l} F_{sc0}=\text{Concat}\left(F_{c},S_{c}\right) \end{array}$$ (6)

Where *S*_*c*_ and *F*_*c*_ denote the final output features of the spatial domain and frequency domain encoder, respectively.

Building on this, channel attention (CA) and spatial attention (SA) are introduced to jointly modulate the fused features, thereby modeling both spatial salience and channel importance simultaneously. Unlike serial attention, these two attention mechanisms act in parallel on the feature representation, enabling synchronous response modeling across both the channel and spatial dimensions. Their computation follows the CBAM (38). After element-wise weighting, a residual connection is introduced to complete the first stage of feature enhancement, as shown in Equation 7:

$$\begin{array}{l} F_{sc1}=P\left(F_{sc0}\right)+CA\left(F_{sc0}\right)⊙SA\left(F_{sc0}\right)⊙F_{sc0} \end{array}$$ (7)

Subsequently, convolutional mapping is applied to compress the features along the channel dimension and reorganize local information, restoring the fused features to a single-branch channel scale, as shown in Equation 8:

$$\begin{array}{l} F_{sc2}=P\left(F_{sc1}\right) \end{array}$$ (8)

Next, attention modulation is applied again to the refined features, and combined with the residual path to form the second-stage enhancement result, as shown in Equation 9:

$$\begin{array}{l} F_{sc3}=P\left(F_{sc2}\right)+CA\left(F_{sc2}\right)⊙SA\left(F_{sc2}\right)⊙F_{sc2} \end{array}$$ (9)

The final output is obtained via a non-linear activation function, as shown in Equation 10:

$$\begin{array}{l} F_{Sc}=\sigma \left(F_{Sc3}\right) \end{array}$$ (10)

Here, CA(·) and SA(·) denote the channel attention and spatial attention operations, respectively. P(·) denotes the convolution mapping process, and σ(·) is the ReLU activation function. PAF achieves progressive optimization through two-stage attention modulation. The resulting fusion is then fed into the decoding path, providing spatial-frequency fusion information for hierarchical recovery and reconstruction with encoded features at different scales.

### 3.4 Frequency Self-Attention Module (FSA)

**Figure 5:**
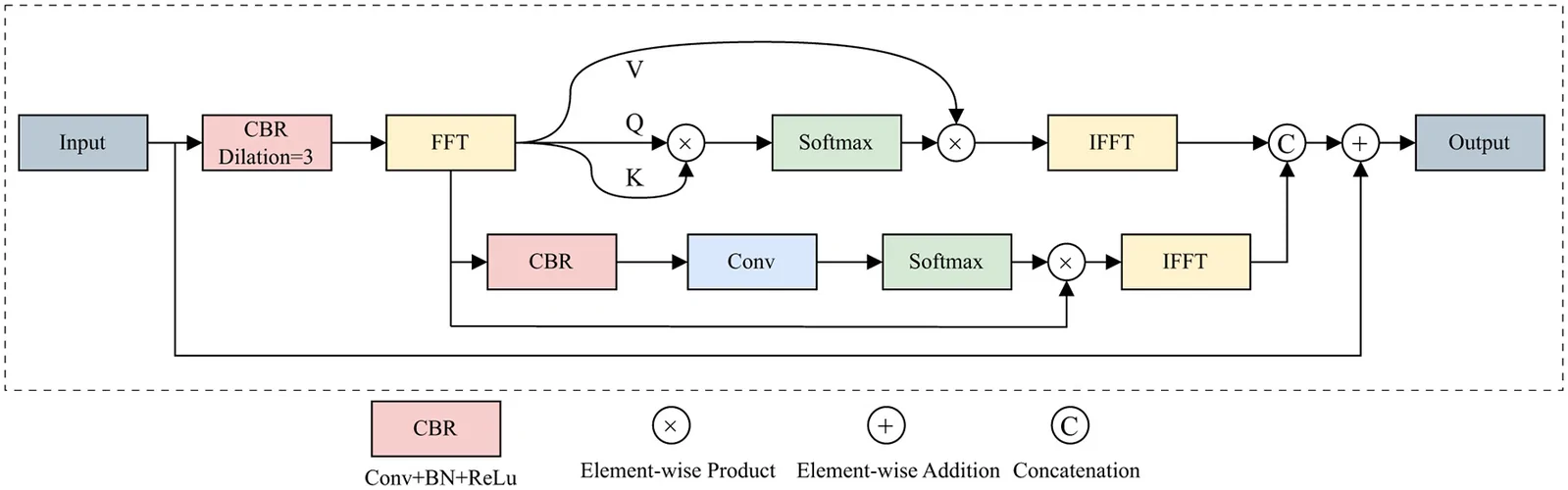
Architecture of the FSA module. Self-attention computations are performed in the frequency domain to model global dependencies among different frequency components.

Spatial features from the encoder primarily represent local structural information but lack sufficient global frequency-dependent characterization. Inspired by Pei et al. (39), we designed the Frequency Self-Attention Module (FSA) to enhance these features in the frequency domain. Based on the frequency representations provided by FreqSep, FSA models the correlations between components in the frequency space and combines self-attention with a frequency-weighting mechanism to amplify key responses. The features processed by the FSA are used in decoding, which helps preserve nodule boundaries and fine-grained structures during the step-by-step reconstruction process, thereby improving the model's ability to distinguish nodule regions. The structure of the FSA is shown in Figure 5.

Given the input feature map *X*, a locally enhanced representation *F* is first obtained via convolution. The feature is then mapped to the frequency domain using the Fast Fourier Transform (FFT) to construct the Query, Key, and Value, as shown in Equation 11:

$$\begin{array}{l} Q,K,V=\text{FFT}\left(F\right) \end{array}$$ (11)

After performing multi-head reshaping and normalization on the frequency-domain features, the frequency-domain attention relationship is computed, where the query and key are dot-producted and scaled using a learnable temperature coefficient τ, as shown in Equation 12:

$$\begin{array}{l} A_{f}^{\prime }=\tilde{Q}{\tilde{K}}^{⊤}\cdot \tau \end{array}$$ (12)

Since $A_{f}^{\prime }$ is a complex number, its real and imaginary parts are normalized and reconstructed separately, as shown in Equation 13:


$$\begin{array}{l} A_{f}^{re}=\frac{A_{f}^{\prime }+\text{conj}\left(A_{f}^{\prime }\right)}{2},\;A_{f}^{im}=\frac{A_{f}^{\prime }-\text{conj}\left(A_{f}^{\prime }\right)}{2i}, \end{array}$$ (13)

$$A_{f}=\Theta \left(\text{Sof}\left(A_{f}^{re}\right),\text{Sof}\left(A_{f}^{im}\right)\right)$$

Here, Θ(·) denotes the function that recombines the real and imaginary parts into a complex form, Sof(·) is used to normalize the attention weights, and *A*_*f*_ is the final attention after complex Softmax normalization.

Next, the frequency-domain attention is weighted with Value and projected back to the spatial domain using IFFT, as shown in Equation 14:

$$\begin{array}{l} F_{g}=|\text{IFFT}\left(A_{f}\cdot V\right)| \end{array}$$ (14)

At the same time, a local enhancement branch based on frequency-domain weight generation is introduced, as shown in Equation 15:


$$\begin{array}{l} F_{l}=|\text{IFFT}\left(W\left(F_{f}^{re}\left(\text{FFT}\left(F\right)\right)\right)⊙\text{FFT}\left(F\right)\right)| \end{array}$$ (15)

where *W*(·) denotes the weight function generated from the frequency-domain features, and *F*_*fre*_(·) denotes the real part of the frequency-domain features.

Finally, the global frequency-domain attention features are concatenated with the local enhancement features, and a residual connection is added to obtain the output, as shown in Equation 16:

$$\begin{array}{l} Y=X+\text{Concat}\left(F_{g},F_{l}\right) \end{array}$$ (16)

### 3.5 Loss functions

Dice loss and binary cross-entropy (BCE) loss are commonly employed as objective functions in medical image segmentation. Dice loss evaluates the overlap between the predicted segmentation and the ground-truth region, making it more responsive to foreground targets and more effective in handling class imbalance during target identification. In contrast, the BCE loss quantifies the difference between predicted probabilities and ground-truth labels at the pixel level, providing a stable optimization signal for each pixel and thereby enhancing the stability of the training process. In tasks such as lung nodule segmentation, where class distribution is imbalanced and target regions are small, combining these two losses creates a synergistic effect.

In this paper, a weighted fusion approach is utilized for loss function construction by linearly integrating Dice loss with BCE loss. Specifically, we assign different weighting coefficients to the two losses and obtain the final optimization objective function through a weighted sum, as shown in Equations 17–19:

$$\begin{array}{l} L=\lambda_{1}\cdot L_{BCE}+\lambda_{2}\cdot L_{Dice} \end{array}$$ (17)

$$\begin{array}{l} L_{BCE}=-\frac{1}{N}\overset{N}{\underset{i=1}{\sum }}\left[y_{i}\log\left(p_{i}\right)+\left(1-y_{i}\right)\log\left(1-p_{i}\right)\right] \end{array}$$ (18)

$$\begin{array}{l} L_{Dice}=1-\frac{2\overset{N}{\underset{i=1}{\sum }}\left(p_{i}\cdot y_{i}\right)+\epsilon }{\overset{N}{\underset{i=1}{\sum }}p_{i}+\overset{N}{\underset{i=1}{\sum }}y_{i}+\epsilon } \end{array}$$ (19)

Here, *p*_*i*_ represents the predicted value for the *i*-th pixel, and *y*_*i*_ indicates the corresponding ground-truth label. *N* indicates the total number of image pixels, and ϵ is a small constant assigned as 1 × 10^−5^ to avoid numerical instability resulting from division by zero. The weight parameters λ_1_ and λ_2_ control the contribution ratios of the BCE loss and Dice loss to the total loss, respectively. In our experiments, we set these weights to 0.5 and 1, respectively, to balance the influence of the two loss functions. The results of different weight combinations are demonstrated in subsequent experiments.

## 4 Experiments

### 4.1 Datasets

Comprehensive experiments were performed on the public LIDC-IDRI dataset and the private FAHGMU-LUNA dataset to assess the performance and effectiveness of the proposed method.

**Figure 6:**
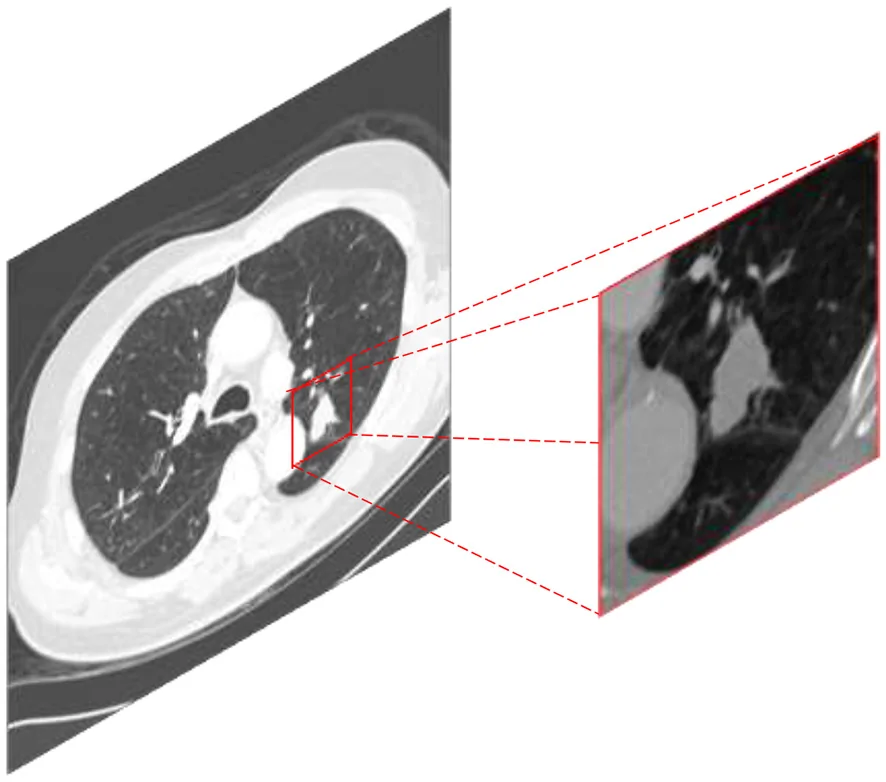
Example of dataset processing: the left panel presents the original CT slice from the LIDC-IDRI dataset, whereas the right panel shows the cropped local region centered around the nodule, including both the nodule itself and the surrounding tissue structures.

LIDC-IDRI Dataset (40): Curated and released by the National Cancer Institute of the United States, this dataset serves as a standardized public repository of lung CT images for studies related to lung cancer and associated lesions. It contains 1,018 chest CT scans acquired from 1,010 patients across multiple medical centers, comprising a total of 15,096 images. Each case was independently reviewed and annotated by four experienced thoracic radiologists, providing detailed annotation information including the location, size, morphology, and density of lung nodules. As shown in Figure 6, given that lung nodules typically occupy a small proportion of CT images, this study retained only axial slices containing nodules based on expert annotations. The annotations from multiple physicians were then fused using a logical OR operation to generate a unified nodule mask. Based on this, the images were cropped with the nodule region as the center and uniformly resized to 128 × 128 pixels. This dataset can be accessed via Link1.

FAHGMU-LUNA Dataset: Provided by the Department of Medical Imaging at the Fourth Affiliated Hospital of Guangxi Medical University, this dataset comprises a total of 1,610 lung CT slices. All CT images have a native resolution of 512–512, were acquired using standard imaging protocols, and were captured using multi-detector spiral CT scanners. The samples originate from different patients and various scanning devices, resulting in variations in scanning angles, noise levels, and image quality. Furthermore, the nodules in this dataset are predominantly small in size and are obscured by complex lung parenchymal textures, which makes the segmentation task more challenging to some extent. During the data preprocessing stage, the original DICOM files were initially converted into PNG format using MicroDICOM software. Subsequently, multiple radiologists used the LabelMe tool to perform mask-based annotation of lung nodules. All cases were independently annotated by three experienced radiologists and were subsequently subjected to final review and quality control by a senior radiologist. To evaluate annotation consistency, inter-observer agreement analysis was performed, and the Dice Similarity Coefficient (DSC) was used for quantitative assessment, demonstrating a high level of consistency among observers. For cases with discrepant annotations, consensus was first reached through group discussion; if agreement could not be achieved, final adjudication was performed by the senior radiologist; samples for which consensus could not be reached were excluded. The entire annotation process underwent multiple rounds of cross-checking and validation to ensure annotation accuracy and consistency. In the subsequent processing stage, the images were cropped around the nodule-centered regions and uniformly resized to a resolution of 128–128. The specific implementation procedures are consistent with (Figure 6).

**Figure 7:**
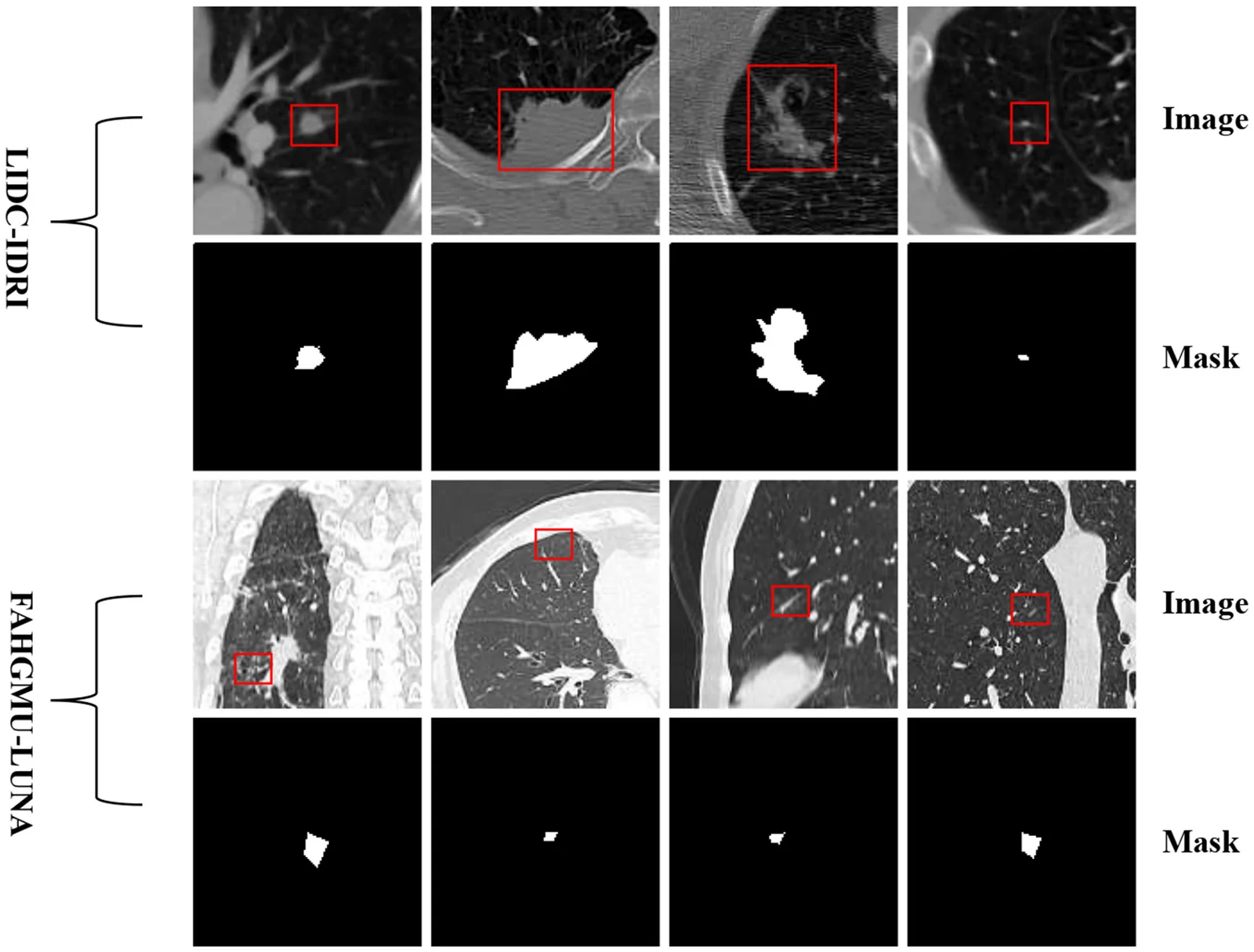
Sample examples of the two lung nodule datasets used in the experiment.

The experimental setup involved splitting 15,096 images from the public LIDC-IDRI dataset into training, validation, and test sets in a 6:2:2 proportion, corresponding to 9,057, 3,019, and 3,020 images, respectively. Likewise, the 1,610 CT images in the FAHGMU-LUNA dataset were split using the same strategy, resulting in 966 training images, 322 validation images, and 322 test images. It should be emphasized that, to avoid data leakage, all dataset splits were performed strictly at the patient level, ensuring that no CT images or nodules from the same patient were split across different subsets. During model training, the optimal model parameters were selected and preserved according to validation set performance, while the final evaluation was conducted on the test set. Figure 7 provides partial examples from both datasets, showcasing representative images of different types of pulmonary nodules.

### 4.2 Implementation details

In this study, we performed a series of preprocessing operations on images, including intensity normalization, bias field correction and noise suppression. Furthermore, all input images were augmented with horizontal flipping, random rotation and random brightness adjustment. These operations were employed to simulate diverse situations that may arise in clinical settings. Specifically, images were horizontally flipped with a probability of *p* = 0.5, brightness and contrast were randomly perturbed within a range of ±0.10, Gaussian noise with a mean of 0 and a variance range of [5, 25] was added, and the images were normalized to the range of [0, 1] using a mean value of 0.2 and a standard deviation of 0.15. Importantly, all augmentation operations were applied synchronously to both the original images and the corresponding ground-truth masks. Each enhancement method was applied independently to the corresponding image patches. All experiments were conducted in a consistent environment using an NVIDIA GeForce RTX 3060 (12GB) graphics card. The models were built in a Python 3.8 environment and implemented using the PyTorch 2.4.1 framework. During training, the following hyperparameter settings were used for both datasets: the optimizer was Adam, training was performed for 100 epochs with an initial learning rate of 5 × 10^−5^, employing a cosine-based learning rate decay strategy, with the learning rate gradually decreasing to 5 × 10^−6^ per epoch, the batch size was 8, the weight decay was 1 × 10^−4^, and the momentum parameter was 0.9. All experiments were conducted with five independent runs using different random seeds, and the results are reported as mean ± standard deviation. Additionally, to prevent model overfitting, an early stopping strategy was applied during training: when the loss value failed to further decrease for 50 successive epochs, the training procedure was stopped in advance.

### 4.3 Evaluation metrics

IoU, Dice coefficient, the 95th percentile Hausdorff distance (HD95) and *F*_β_-score (β = 2, 0.5) are selected as evaluation metrics in this paper to comprehensively evaluate model performance.

The Hausdorff distance is used to quantify the spatial distance discrepancy between the predicted segmentation boundary and the ground truth boundary. A lower HD95 value indicates a higher degree of consistency between the predicted boundary and the ground truth boundary. The calculation formula is shown in Equation 20:

$$\begin{array}{l} \text{HD}95=P_{95}\left(\left\{\underset{y\in Y}{\min}d\left(x,y\right)\right\}\cup \left\{\underset{x\in X}{\min}d\left(y,x\right)\right\}\right) \end{array}$$ (20)

Where *X* and *Y* denote the boundary point sets of the ground truth and predicted segmentation, respectively. *x* and *y* represent the points on the corresponding boundaries, and *d*(*x, y*) denotes the Euclidean distance between two boundary points. *P*_95_ represents the 95th percentile of the bidirectional boundary distance distribution, which reduces the influence of the largest 5% outlier distances.

IoU reflects the overlap between the predicted segmentation and the ground truth by calculating the ratio between their intersection and union, serving as a key metric for measuring segmentation accuracy, as shown in Equation 21:

$$\begin{array}{l} IoU=\frac{1}{n}\overset{n}{\underset{i=1}{\sum }}\frac{TP_{i}}{TP_{i}+FP_{i}+FN_{i}} \end{array}$$ (21)

The Dice coefficient is employed to evaluate the overlap between the predicted segmentation and the ground-truth annotations, assigning higher weights to overlapping regions, making it more sensitive when evaluating segmentation performance, as shown in Equation 22:

$$\begin{array}{l} Dice=\frac{1}{n}\overset{n}{\underset{i=1}{\sum }}\frac{2\cdot TP_{i}}{2\cdot TP_{i}+FP_{i}+FN_{i}} \end{array}$$ (22)

*F*_β_-score combines information from precision and recall, using the parameter β to adjust their relative influence, thereby adapting to different task requirements. When β>1, the evaluation favors recall, highlighting the model's ability to comprehensively identify target regions. And when β < 1, the evaluation emphasizes precision, focusing on reducing the proportion of false predictions, as shown in Equation 23:

$$\begin{array}{l} F_{\beta }=\frac{\left(1+\beta^{2}\right)\cdot \text{precision}\cdot \text{recall }}{\beta^{2}\cdot \text{precision}+\text{recall}},\\ \text{ precision}=\frac{1}{n}\sum_{i=1}^{n}\frac{TP_{i}}{TP_{i}+FP_{i}},\\ \text{ recall}=\frac{1}{n}\sum_{i=1}^{n}\frac{TP_{i}}{TP_{i}+FP_{i}} \end{array}$$ (23)

In Equations 20–22, *TP*_*i*_, *FP*_*i*_, and *FN*_*i*_ represent the true positive, false positive, and false negative regions for class *i*, respectively, and *n* denotes the number of classes. These metrics characterize the model's performance in the lung nodule segmentation task from different perspectives and together form a comprehensive evaluation framework.

### 4.4 Comparative experiments

To comprehensively evaluate the segmentation capability of the proposed DD-Net, comparative experiments with multiple models were performed on the LIDC-IDRI and FAHGMU-LUNA datasets. These included the classic UNet (41), the CNN-Transformer hybrid models UCTransNet (21), TransUnet (22), and BRAU-Net++ (23), the dual-domain model SF-UNet (29) which integrates frequency-domain information, the dual-attention-based DA-TransUNet (42), and the lightweight M^2^SNet (43). The compared models were implemented based on public open-source code and aligned with our model in terms of data splitting, augmentation pipeline, and hyperparameter settings to guarantee fairness.

#### 4.4.1 Results on the LIDC-IDRI dataset

Table 1 presents the quantitative comparison of different methods on the LIDC-IDRI dataset. The experimental results are reported in the form of mean ± standard deviation, with bold font indicating the optimal value for each evaluation metric. Clearly, our DD-Net method outperforms other representative baselines on all metrics, achieving a best IoU of 88.02% and a best Dice score of 92.51%. Compared to the second-best BRAU-Net++, our method improves IoU by 1.44% and Dice score by 1.02%. Compared to the classic UNet method, IoU increased by 2.23% and the Dice score by 1.23%. Additionally, our method achieved optimal mean values of 92.68% and 93.05% for F2-score and F0.5-score, respectively, and obtained the lowest HD95 value, demonstrating dual advantages in both the precise identification of lung nodules and the delineation of their boundaries.

**Table 1:** Comparison of DD-Net and other methods on the LIDC-IDRI dataset.

Method	Source	IoU (%)	Dice (%)	HD95	F2-score (%)	F0.5-score (%)
UNet (41)	MICCAI (2015)	85.79 ± 0.230	91.28 ± 0.143	2.19 ± 0.047	91.28 ± 0.140	92.06 ± 0.143
UCTransNet (21)	AAAI (2022)	85.91 ± 0.140	91.36 ± 0.080	2.10 ± 0.019	91.48 ± 0.148	92.03 ± 0.101
M^2^SNet (43)	TMI (2023)	83.12 ± 0.131	89.30 ± 0.134	2.52 ± 0.031	90.11 ± 0.125	89.71 ± 0.129
TransUnet (22)	MIA (2024)	85.38 ± 0.076	90.81 ± 0.057	2.29 ± 0.043	90.96 ± 0.039	91.57 ± 0.099
DA-TransUNet (42)	FIBB (2024)	84.31 ± 0.130	90.13 ± 0.067	2.41 ± 0.004	91.21 ± 0.089	91.07 ± 0.130
SF-UNet (29)	BIBM (2024)	86.51 ± 0.125	91.42 ± 0.128	2.04 ± 0.030	91.53 ± 0.115	92.29 ± 0.104
BRAU-Net++ (23)	IEEE (2026)	86.58 ± 0.149	91.49 ± 0.124	2.06 ± 0.024	91.57 ± 0.125	92.19 ± 0.112
DD-Net (ours)	–	**88.02 ±0.064**	**92.51 ±0.052**	**1.10 ±0.021**	**92.68 ±0.030**	**93.05 ±0.091**

The experimental results are reported in the form ofmean 1 standard deviation, with bold font indicating the optimal value for each evaluation metric.

**Figure 8:**
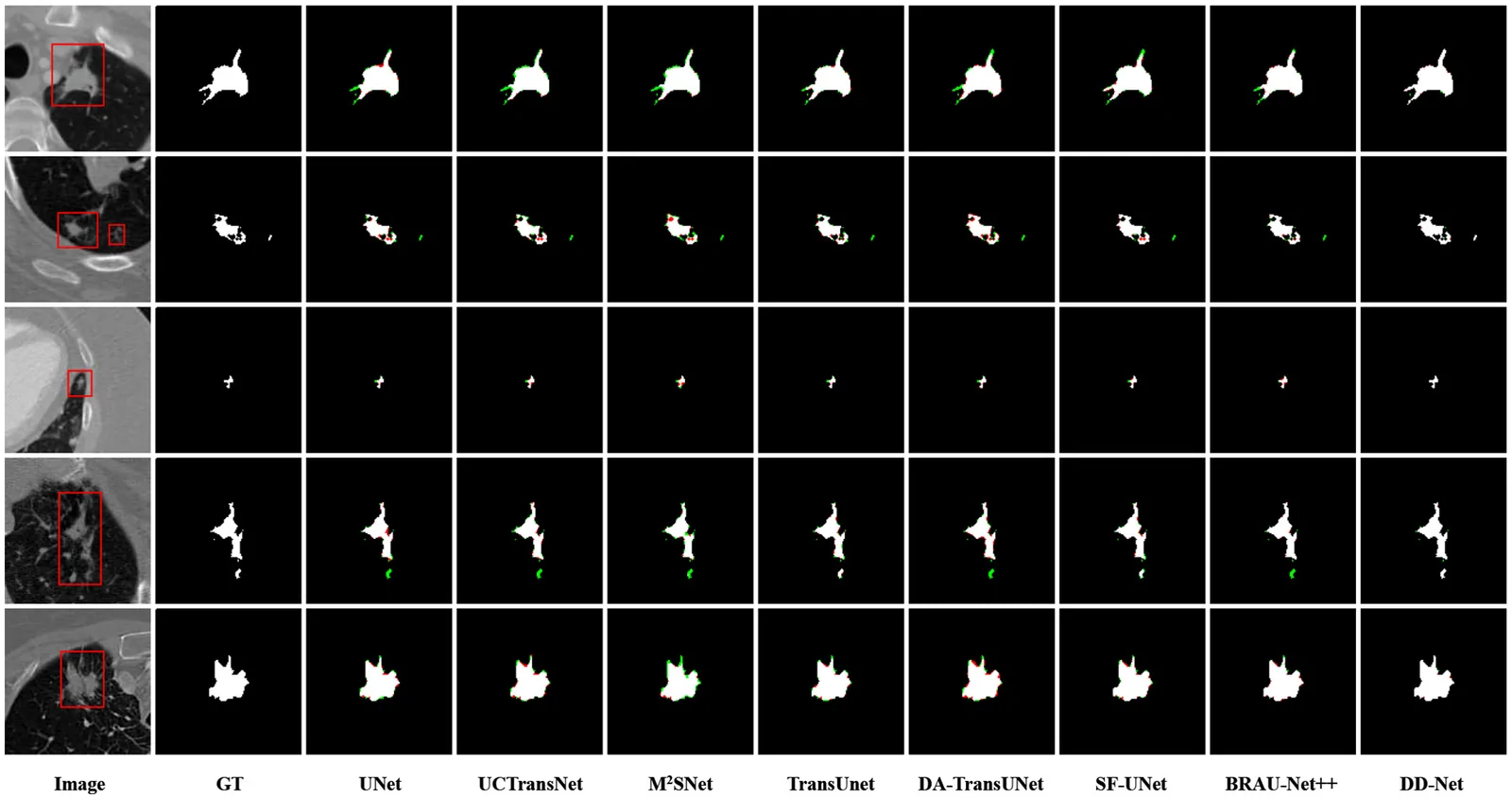
Visual comparison of different segmentation models on the LIDC-IDRI dataset. The white regions indicate correctly segmented lesions, the red regions denote false positive predictions, and the green regions correspond to false negative predictions.

Figure 8 illustrates the qualitative comparison results obtained on the LIDC-IDRI dataset. It can be observed that the proposed DD-Net exhibits stronger segmentation performance even under complex background conditions. For example, in the first row, the lesion boundary is irregular and fused with surrounding tissue. UNet, DA-TransUNet, and SF-UNet still exhibit significant errors along the upper and lower edges, while UCTransNet, M^2^SNet, TransUnet, and BRAU-Net++ also show scattered deviations. In contrast, DD-Net maintains a more complete contour.In the second row, the lesion is irregular with blurred boundaries. UNet, UCTransNet, M^2^SNet, and SF-UNet all exhibit significant missed and false-positive detections, while TransUnet, DA-TransUNet, and BRAU-Net++ show sporadic missed detections. In contrast, DD-Net achieves nearly error-free segmentation results. In the third row, a very small lesion is embedded within a complex structure. Misclassifications are most pronounced in BRAU-Net++, TransUnet, and M^2^SNet, with both red and green artifacts being quite noticeable. UCTransNet and SF-UNet also exhibit slight deviations, while DD-Net remains capable of reliably identifying the target.In the fourth row, the elongated structure increases segmentation difficulty. Most methods exhibit missed detections in the lower extension area, failing to maintain continuity for such elongated targets, whereas DD-Net still achieves a relatively complete segmentation. In the fifth row, the background texture is complex and the edges exhibit some complexity. UCTransNet, M^2^SNet, and DA-TransUNet all show noticeable errors, and other methods also exhibit some errors, whereas DD-Net closely aligns with the ground truth. This indicates that the proposed method can effectively cope with the challenges posed by diverse nodule morphologies and complicated background structures in the LIDC-IDRI dataset, demonstrating more stable and accurate lung nodule segmentation capabilities.

Furthermore, we compared our method with various other approaches on the same lung nodule segmentation task, with the results shown in Table 2. The results of the compared methods in the table are based on the LIDC-IDRI dataset and are all cited from the original papers. The best values for each metric are highlighted in bold. The experimental results for DD-Net are presented as mean ± standard deviation. It can be seen that DD-Net achieves the best performance across all key metrics, with mean IoU and Dice scores reaching 88.02% and 92.51%, respectively, outperforming all comparison methods in overall performance. Compared to Cai et al. (18), IoU and Dice improved by 7.24% and 3.32%, respectively. Compared to Li et al. (5), IoU improved by 2.65% and Dice by 0.45%. Compared to Abulizi et al. (19), IoU improved by 3.51% and Dice by 0.69%. For Tang et al. (17), which reported only Dice, our method also achieved higher values. The results demonstrate that DD-Net possesses stronger advantages in both region overlap and segmentation consistency, resulting in more stable and reliable segmentation performance.

**Table 2:** Comparison of experimental results for the same lung nodule segmentation task.

Existing deep learning related work	IoU (%)	Dice (%)
Tang et al. (17)	–	88.90
Cai et al. (18)	80.78	89.19
Li et al. (5)	85.37	92.06
Abulizi et al. (19)	84.51	91.82
Proposed DD-Net	**88.02 ±0.064**	**92.51 ±0.052**

The experimental results are reported in the form ofmean 1 standard deviation, with bold font indicating the optimal value for each evaluation metric.

#### 4.4.2 Results on the FAHGMU-LUNA dataset

Table 3 reports the quantitative comparison results of different methods on the FAHGMU-LUNA dataset. The experimental results are reported in the form of mean ± standard deviation, with bold font indicating the optimal value for each evaluation metric. The comparison reveals that DD-Net achieves the best results on this dataset, with mean values of 65.03% for IoU, 77.08% for Dice, 79.34% for F2-score, and 77.62% for F0.5-score, together with improved boundary delineation performance measured by HD95, outperforming all compared methods overall. Compared to SF-UNet, which also incorporates frequency-domain information, DD-Net achieves improvements of 1.74% and 1.00% in IoU and Dice, respectively, and 2.97% in F2-score, striking a better balance between precision and recall. It can also be observed that the overall performance on this dataset is lower than that on LIDC-IDRI, primarily due to the generally smaller nodule sizes and the superimposed interference from large areas of complex lung parenchyma texture.

**Table 3:** Comparison of DD-Net and other methods on the FAHGMU-LUNA dataset.

Method	Source	IoU (%)	Dice (%)	HD95	F2-score (%)	F0.5-score (%)
UNet (41)	MICCAI (2015)	63.28 ± 0.383	75.18 ± 0.864	3.89 ± 0.161	74.76 ± 0.193	77.25 ± 0.569
UCTransNet (21)	AAAI (2022)	61.61 ± 0.328	73.99 ± 0.882	4.01 ± 0.078	73.11 ± 0.397	77.47 ± 0.321
M^2^SNet (43)	TMI (2023)	54.39 ± 0.245	68.05 ± 0.723	4.48 ± 0.116	67.44 ± 0.484	71.08 ± 0.330
TransUnet (22)	MIA (2024)	55.82 ± 0.376	69.81 ± 0.354	3.88 ± 0.094	68.26 ± 0.453	73.96 ± 0.515
DA-TransUNet (42)	FIBB (2024)	56.55 ± 0.590	69.83 ± 0.751	4.32 ± 0.110	68.70 ± 1.011	74.25 ± 0.710
SF-UNet (29)	BIBM (2024)	63.29 ± 0.269	76.08 ± 0.412	3.71 ± 0.074	76.37 ± 0.814	75.48 ± 0.613
BRAU-Net++ (23)	IEEE (2026)	59.80 ± 0.393	73.15 ± 0.284	4.13 ± 0.080	72.98 ± 0.670	75.95 ± 0.606
DD-Net (ours)	-	**65.03 ±0.217**	**77.08 ±0.301**	**2.91 ±0.064**	**79.34 ±0.278**	**77.62 ±0.346**

The experimental results are reported in the form ofmean 1 standard deviation, with bold font indicating the optimal value for each evaluation metric.

**Figure 9:**
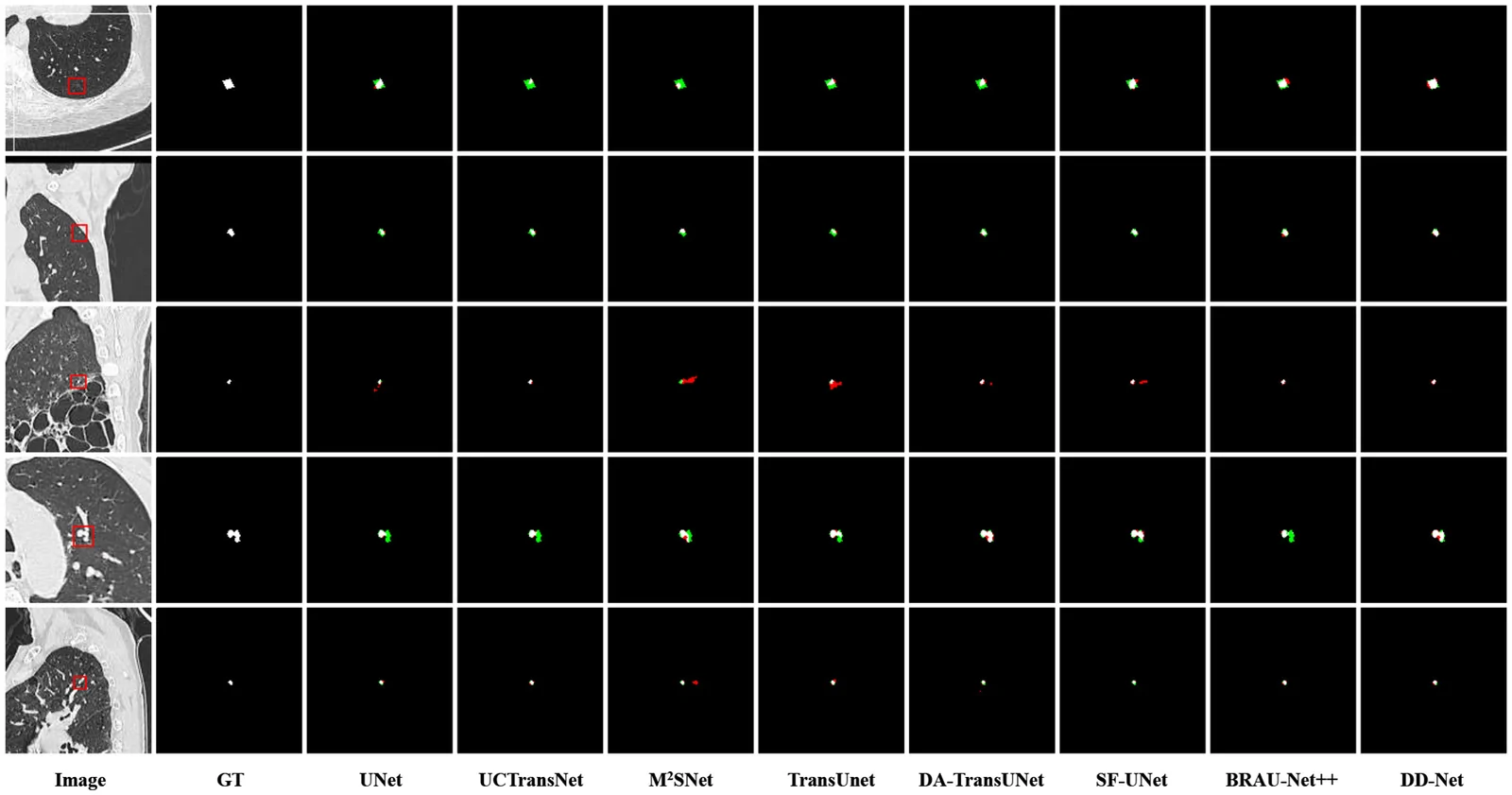
Visual comparison of different segmentation models on the FAHGMU-LUNA dataset. The white regions indicate correctly segmented lesions, the red regions denote false positive predictions, and the green regions correspond to false negative predictions.

The qualitative experimental results for the FAHGMU-LUNA dataset are shown in Figure 9. Overall, the performance differences of various algorithms are more pronounced in complex backgrounds. For example, in the first row, UCTransNet, M^2^SNet, TransUnet, and DA-TransUNet exhibit varying degrees of under-detection at the lesion edges, while UNet and SF-UNet are accompanied by a small number of false positives. DD-Net is able to preserve the target contours more completely.In the second row, BRAU-Net++ and SF-UNet still exhibit some red false positives around the target, while M^2^SNet shows minor omissions. DD-Net's results align more closely with the ground truth.The differences are most pronounced in the third row: M^2^SNet and TransUnet exhibit significant missegmentation with numerous red false positives, while DA-TransUNet and SF-UNet show scattered deviations. In contrast, DD-Net maintains cleaner and more stable segmentation results. In the fourth row, DA-TransUNet and UNet still exhibit omissions at local boundaries, while UCTransNet and BRAU-Net++ lack sufficient detail recovery. DD-Net, however, better preserves fine-grained structures. In the fifth row, M^2^SNet and TransUnet exhibit significant deviations, and the remaining methods also show varying degrees of boundary distortion, whereas DD-Net remains closest to the ground truth. Overall, compared to the comparison methods, DD-Net effectively reduces false positive and false negative regions under complex background and extremely small object conditions, demonstrating stronger robustness and detail rendering capabilities.

### 4.5 Ablation studies

#### 4.5.1 Module ablation study

To validate the effectiveness of each component, we used a pre-trained ResNet34 as the encoder and Resblock with Upsample as the basic decoder unit, while retaining standard skip connections and feature concatenation to construct the baseline model.Based on this, we designed and conducted the following ablation experiments: Baseline, Baseline + FreqSep module, Baseline + FSA module, Baseline + FreqSep + PAF module, Baseline + FreqSep + FSA module, and Baseline + FreqSep + PAF + FSA module (DD-Net). To ensure experimental fairness, all models used consistent hyperparameter settings during training, such as learning rate and batch size. The ablation experiments were conducted on the LIDC-IDRI and FAHGMU-LUNA datasets, using IoU, Dice, HD95, F2-score, and F0.5-score as evaluation metrics.

**Figure 10:**
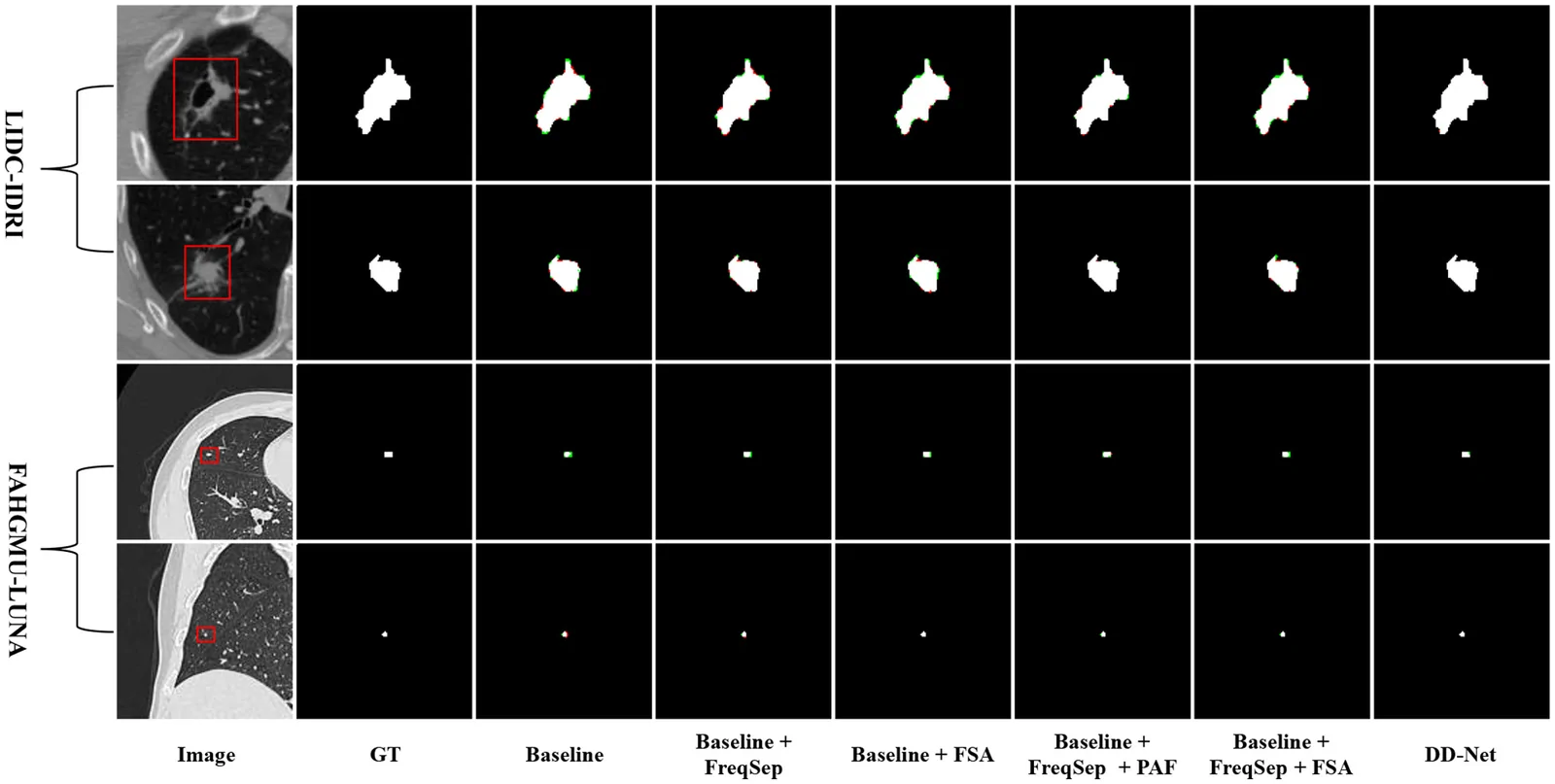
Visualization results of the module ablation experiments. The white regions indicate correctly segmented lesions, the red regions denote false positive predictions, and the green regions correspond to false negative predictions.

Table 4 shows the ablation study results of each module on the two datasets. The experimental results are reported in the form of mean ± standard deviation, with bold font indicating the optimal value for each evaluation metric. First, the FreqSep module was integrated into the baseline. Compared to the baseline, FreqSep increased IoU and Dice by 1.18% and 0.77%, respectively, on the LIDC-IDRI dataset, and by 0.57% and 0.34%, respectively, on the FAHGMU-LUNA dataset, indicating that frequency-domain separation has a positive effect on feature representation. When only the FSA module was introduced, it also yielded consistent gains on both datasets. Further combining the FreqSep module with the PAF module continues to improve model performance, achieving an IoU of 87.76% and a Dice score of 92.31% on the LIDC-IDRI dataset, and an IoU of 64.72% and a Dice score of 76.92% on the FAHGMU-LUNA dataset. Meanwhile, combining FreqSep with FSA also leads to continuous improvements in all metrics. It can be observed that each component module improved the baseline model to varying degrees. Ultimately, through the synergistic interaction of the three modules, the model achieved optimal results across all evaluation metrics. Compared to the baseline, IoU and Dice improved by 1.83% and 0.98%, respectively, on LIDC-IDRI, and by 1.66% and 1.27%, respectively, on FAHGMU-LUNA, with other metrics also showing corresponding improvements. The visualization results of the module ablation experiments are shown in Figure 10. The results indicate that the synergy among the modules brings stable performance gains to the model, fully validating the effectiveness and necessity of the component designs.

**Table 4:** Results of module ablation experiments.

Dataset	IoU (%)	Dice (%)	HD95	F2-score (%)	F0.5-score (%)	FreqSep	PAF	FSA
LIDC-IDRI	86.19 ± 0.091	91.53 ± 0.088	1.86 ± 0.042	91.74 ± 0.095	92.15 ± 0.134	×	×	×
	87.37 ± 0.106	92.30 ± 0.085	1.39 ± 0.034	92.24 ± 0.093	92.69 ± 0.121	✓	×	×
	87.24 ± 0.069	92.12 ± 0.074	1.45 ± 0.032	92.21 ± 0.075	92.70 ± 0.098	×	×	✓
	87.76 ± 0.063	92.31 ± 0.067	1.32 ± 0.030	92.38 ± 0.086	92.81 ± 0.115	✓	✓	×
	87.72 ± 0.074	92.45 ± 0.061	1.28 ± 0.027	92.42 ± 0.074	92.76 ± 0.117	✓	×	✓
	**88.02 ±0.064**	**92.51 ±0.052**	**1.10 ±0.021**	**92.68 ±0.030**	**93.05 ±0.091**	✓	✓	✓
FAHGMU-LUNA	63.37 ± 0.351	75.81 ± 0.578	3.64 ± 0.071	77.57 ± 0.346	75.94 ± 0.395	×	×	×
	63.94 ± 0.346	76.15 ± 0.416	3.47 ± 0.082	77.92 ± 0.373	76.54 ± 0.378	✓	×	×
	63.79 ± 0.255	76.13 ± 0.651	3.51 ± 0.077	77.84 ± 0.496	76.51 ± 0.601	×	×	✓
	64.72 ± 0.311	76.92 ± 0.359	3.14 ± 0.065	78.90 ± 0.455	77.44 ± 0.481	✓	✓	×
	64.67 ± 0.243	76.87 ± 0.308	3.19 ± 0.069	79.06 ± 0.511	77.37 ± 0.377	✓	×	✓
	**65.03 ±0.217**	**77.08 ±0.301**	**2.91 ±0.064**	**79.34 ±0.278**	**77.62 ±0.346**	✓	✓	✓

The experimental results are reported in the form ofmean 1 standard deviation, with bold font indicating the optimal value for each evaluation metric.

#### 4.5.2 Ablation experiments on channel separation in the FreqSep

In the FreqSep module, the input feature map *G* is initially divided along the channel dimension according to the ratio α into a high-frequency component *G*_1_ and a low-frequency component *G*_2_. This channel splitting is the core operation of the FreqSep module, effectively separating features that the model needs to process across different frequency domains. We conducted ablation experiments on the LIDC-IDRI and FAHGMU-LUNA datasets to determine the optimal channel splitting ratio and analyze its impact on lung nodule segmentation performance. Table 5 presents the ablation results for channel splitting in the FreqSep module. All results are reported in the form of mean ± standard deviation, with bold font indicating the optimal value for each evaluation metric. When the channel allocation ratio was set to 7:3, the model achieved optimal performance across all evaluation metrics on both test datasets. Other ratio configurations, such as 2:8 and 9:1, while capable of separating high- and low-frequency features, resulted in an imbalanced feature distribution that failed to adequately capture the characteristic feature distribution of both the edge details and global structure of lung nodules. This paper ultimately adopted the optimal 7:3 ratio for channel partitioning, with the signals fed into high-frequency and low-frequency sub-branches for frequency-domain filtering and feature enhancement, respectively. This design allows high-frequency information associated with fine-grained lung nodule details to receive refined frequency-domain enhancement, while low-frequency information related to the global structure and semantic characteristics of the lesion can be sufficiently modeled and strengthened, thereby enabling effective collaboration between the two feature domains.

**Table 5:** Ablation experiment results for channel partitioning in the FreqSep module.

G_1_:G_2_	LIDC-IDRI					FAHGMU-LUNA				
	IoU (%)	Dice (%)	HD95	F2-score (%)	F0.5-score (%)	IoU (%)	Dice (%)	HD95	F2-score (%)	F0.5-score (%)
1:9	87.65 ± 0.078	92.28 ± 0.057	1.42 ± 0.023	92.46 ± 0.031	92.84 ± 0.099	64.37 ± 0.219	76.42 ± 0.339	3.30 ± 0.084	79.25 ± 0.301	77.54 ± 0.374
2:8	87.45 ± 0.067	92.17 ± 0.049	1.46 ± 0.030	92.40 ± 0.042	92.68 ± 0.102	64.51 ± 0.234	76.47 ± 0.361	3.23 ± 0.066	79.23 ± 0.299	77.48 ± 0.355
3:7	87.34 ± 0.071	91.74 ± 0.063	1.67 ± 0.048	92.21 ± 0.052	92.45 ± 0.113	64.22 ± 0.258	76.31 ± 0.318	3.36 ± 0.075	79.14 ± 0.287	77.54 ± 0.403
4:6	87.63 ± 0.086	92.28 ± 0.070	1.45 ± 0.029	92.56 ± 0.057	92.73 ± 0.108	64.31 ± 0.233	76.48 ± 0.326	3.31 ± 0.083	78.96 ± 0.311	77.36 ± 0.369
5:5	87.59 ± 0.080	92.23 ± 0.067	1.46 ± 0.031	92.35 ± 0.039	92.83 ± 0.097	64.90 ± 0.315	76.88 ± 0.316	3.16 ± 0.072	78.89 ± 0.325	77.39 ± 0.371
6:4	87.82 ± 0.068	92.06 ± 0.062	1.49 ± 0.026	92.36 ± 0.045	92.58 ± 0.095	64.39 ± 0.288	76.70 ± 0.342	3.19 ± 0.078	79.21 ± 0.279	77.40 ± 0.394
7:3	**88.02 ± 0.064**	**92.51 ± 0.052**	**1.10 ±**
**0.021**	**92.68 ± 0.030**	**93.05 ± 0.091**	**65.03 ± 0.217**	**77.08 ± 0.301**	**2.91 ±**
**0.064**	**79.34 ± 0.278**	**77.62 ± 0.346**
8:2	87.76 ± 0.073	92.05 ± 0.074	1.51 ± 0.033	92.38 ± 0.050	92.54 ± 0.114	64.82 ± 0.249	76.41 ± 0.345	3.35 ± 0.067	79.14 ± 0.304	77.55 ± 0.361
9:1	87.45 ± 0.076	92.20 ± 0.068	1.43 ± 0.027	92.40 ± 0.046	92.73 ± 0.102	64.33 ± 0.256	76.05 ± 0.336	3.50 ± 0.080	79.18 ± 0.289	77.32 ± 0.355

The experimental results are reported in the form ofmean 1 standard deviation, with bold font indicating the optimal value for each evaluation metric.

#### 4.5.3 Ablation study on frequency filter radius

In the frequency domain, the filter screens different frequency components by setting a radius, thereby striking a balance between preserving key information and suppressing redundant noise. To explore the influence of the filtering radius on lung nodule segmentation performance, ablation experiments were performed under different radius settings to identify the optimal parameter for effectively separating high-frequency details from low-frequency global information. The corresponding experimental results are presented in Table 6. The experimental results are reported in the form of mean ± standard deviation, with bold font indicating the optimal value for each evaluation metric. When the filtering radius is set to *H*/8, across the LIDC-IDRI and FAHGMU-LUNA datasets, the model attains the best overall performance for all evaluation metrics. This setting facilitates an effective separation between high-frequency and low-frequency information in the frequency domain, thereby reducing interference and overlap between the two feature components. When the radius is too large, such as at *H*/2, the model overemphasizes the retention of low-frequency global information, causing key local details—such as lung nodule edges and texture—to be filtered out, which directly reduces the model's accuracy in delineating nodule boundaries. Conversely, when the radius is too small, the model becomes overly focused on local details, leading to interference from noise and artifacts, making it difficult to effectively extract the global structural semantics of the lesion, resulting in a decline in key metrics such as IoU and Dice.

**Table 6:** Ablation experiment results for the filtering radius in the FreqSep module.

Filter radius	LIDC-IDRI					FAHGMU-LUNA				
	IoU (%)	Dice (%)	HD95	F2-score (%)	F0.5-score (%)	IoU (%)	Dice (%)	HD95	F2-score (%)	F0.5-score (%)
H/2	87.48 ± 0.067	91.98 ± 0.074	1.57 ± 0.022	92.34 ± 0.033	92.57 ± 0.124	64.85 ± 0.231	76.49 ± 0.321	3.23 ± 0.073	79.17 ± 0.286	77.39 ± 0.357
H/4	87.71 ± 0.051	92.19 ± 0.069	1.50 ± 0.034	92.41 ± 0.048	92.85 ± 0.096	64.83 ± 0.219	76.44 ± 0.314	3.28 ± 0.085	79.11 ± 0.297	77.45 ± 0.354
H/6	87.79 ± 0.057	92.24 ± 0.057	1.46 ± 0.027	92.53 ± 0.042	92.91 ± 0.108	64.79 ± 0.229	76.85 ± 0.318	3.14 ± 0.077	79.23 ± 0.281	77.44 ± 0.363
H/8	**88.02 ± 0.064**	**92.51 ± 0.052**	**1.10 ± 0.021**	**92.68 ± 0.030**	**93.05 ± 0.091**	**65.03 ± 0.217**	**77.08 ± 0.301**	**2.91 ± 0.064**	**79.34 ± 0.278**	**77.62 ± 0.346**
H/10	87.70 ± 0.073	92.16 ± 0.060	1.53 ± 0.026	92.46 ± 0.049	92.73 ± 0.112	64.77 ± 0.225	76.64 ± 0.294	3.20 ± 0.070	79.12 ± 0.284	77.40 ± 0.352

The experimental results are reported in the form ofmean 1 standard deviation, with bold font indicating the optimal value for each evaluation metric.

#### 4.5.4 Ablation analysis of different loss functions

To investigate the differences in the impact of the two loss functions on the lung nodule segmentation task under different weight ratios and to validate the practical application effectiveness of different loss function combinations, this study designed five loss function combination schemes, including standalone BCE loss, standalone Dice loss, and three hybrid combinations of BCE and Dice losses with different weighting ratio. The specific performance of the different loss function combinations is shown in Table 7. The experimental results are reported in the form of mean ± standard deviation, with bold font indicating the optimal value for each evaluation metric. Experimental data show that among the five loss function combinations, the 0.5*BCE+Dice hybrid loss ratio performs best, achieving a mean IoU of 88.02% and a mean Dice coefficient of 92.51% on the LIDC-IDRI dataset, and outperforming other combinations across all evaluation metrics on the FAHGMU-LUNA dataset. This combination effectively integrates the advantages of the BCE loss-such as gradient stability and ease of model convergence-with the characteristics of the Dice loss, which is sensitive to segmentation boundaries and capable of accurately capturing target regions. It not only improves the segmentation accuracy of lung nodule boundary details but also ensures the global stability of model training. Overall, adopting a reasonable weighting strategy between BCE loss and Dice loss can effectively alleviate the class imbalance problem while improving the segmentation accuracy of lung nodules, indicating that the loss function design in this study possesses strong effectiveness and advantages.

**Table 7:** Ablation study on different loss functions.

Loss function	LIDC-IDRI					FAHGMU-LUNA				
	IoU (%)	Dice (%)	HD95	F2-score (%)	F0.5-score (%)	IoU (%)	Dice (%)	HD95	F2-score (%)	F0.5-score (%)
BCE	86.74 ± 0.068	89.64 ± 0.059	2.29 ± 0.019	91.92 ± 0.038	91.77 ± 0.097	63.84 ± 0.228	75.97 ± 0.311	3.69 ± 0.069	77.98 ± 0.286	76.86 ± 0.355
Dice	87.02 ± 0.074	91.97 ± 0.057	1.64 ± 0.028	91.93 ± 0.046	92.72 ± 0.104	64.26 ± 0.224	76.64 ± 0.324	3.15 ± 0.077	78.36 ± 0.255	77.25 ± 0.338
BCE+Dice	87.73 ± 0.065	92.32 ± 0.066	1.44 ± 0.031	92.43 ± 0.036	92.78 ± 0.114	64.87 ± 0.221	76.96 ± 0.316	3.07 ± 0.074	78.86 ± 0.269	77.45 ± 0.358
BCE+0.5Dice	87.34 ± 0.078	92.15 ± 0.064	1.51 ± 0.025	92.35 ± 0.041	92.69 ± 0.107	64.53 ± 0.218	76.84 ± 0.320	3.12 ± 0.068	78.45 ± 0.284	77.31 ± 0.349
0.5BCE+Dice	**88.02 ± 0.064**	**92.51 ± 0.052**	**1.10 ± 0.021**	**92.68 ± 0.030**	**93.05 ± 0.091**	**65.03 ± 0.217**	**77.08 ± 0.301**	**2.91 ± 0.064**	**79.34 ± 0.278**	**77.62 ± 0.346**

The experimental results are reported in the form ofmean 1 standard deviation, with bold font indicating the optimal value for each evaluation metric.

### 4.6 Algorithm complexity

To intuitively compare the computational efficiency of various pulmonary nodule segmentation algorithms, this paper selects floating-point operations (FLOPs) and the number of network parameters (Params) as the core evaluation metrics, and further uses the inference time per image(Inf. Time), peak GPU memory consumption(Peak GPU Mem.), and training time(Train Time) of each model on the LIDC-IDRI dataset as supplementary comparison criteria. The comparison results are shown in Table 8. The proposed method requires 7.69G FLOPs, which is substantially lower compared with methods such as SF-UNet (18.95G FLOPs), UNet (13.67G FLOPs), and UCTransNet (10.80G FLOPs), effectively reducing computational resource consumption and improving operational efficiency.Its parameter count is 37.01M, far lower than that of UCTransNet (66.24M), DA-TransUNet (94.51M), and TransUnet (93.23M), and slightly higher than the lightweight model M^2^SNet (27.69M), achieving a more effective trade-off between model complexity and segmentation performance. It should be further noted that the computational complexity of the proposed method is not optimal, mainly because 12 Frequency Filter Layers are introduced into the frequency-domain encoder, however, the improvement in accuracy makes this acceptable. Overall, there is still room for further improvement in the model's computational efficiency, which will be a focus of our future research.

**Table 8:** Comparison of model computational efficiency.

Method	FLOPs (G)	Params (M)	Peak GPU Mem. (MB)	Train time (h)	Inf. time (ms/img)
UNet (41)	13.67	31.04	1204.3	8.36	9.03
UCTransNet (21)	10.80	66.24	3770.4	12.04	36.52
M^2^SNet (43)	2.26	27.69	1013.9	8.13	16.36
TransUnet (22)	8.05	93.23	2498.5	9.68	19.96
DA-TransUNet (42)	8.29	94.51	2596.4	10.12	19.22
SF-UNet (29)	18.95	28.84	2420.7	11.68	13.94
BRAU-Net++ (23)	5.60	50.76	2073.0	12.82	23.44
DD-Net (ours)	7.69	37.01	1286.7	9.89	18.07

## 5 Discussion

**Figure 11:**
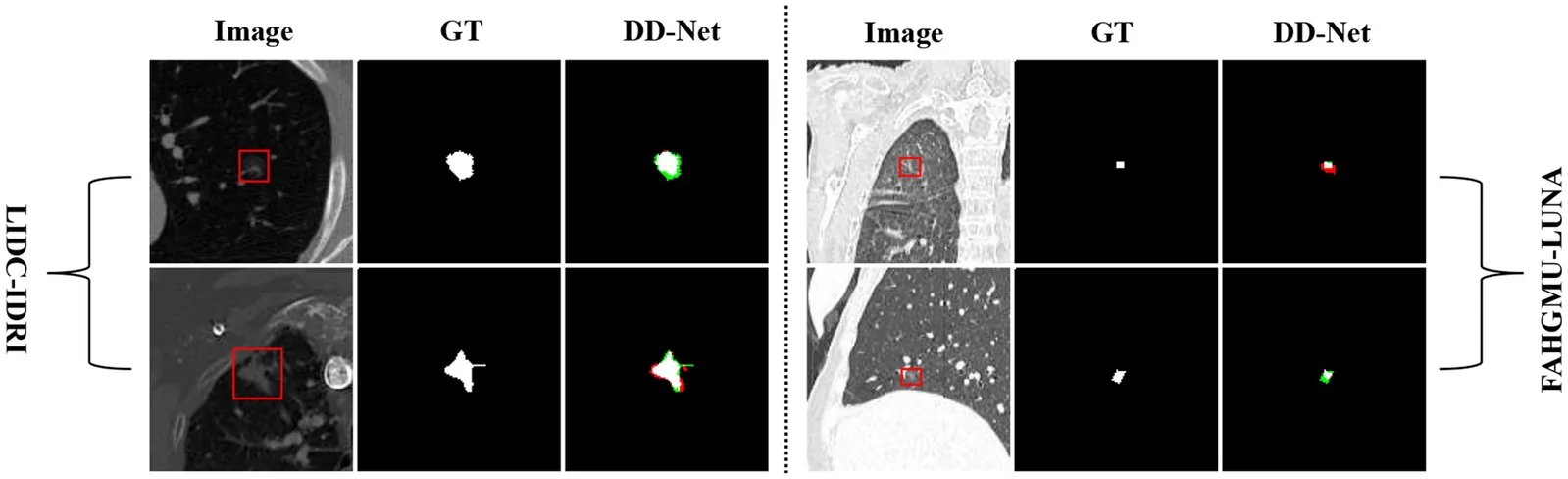
Challenging failure cases. The white regions indicate correctly segmented lesions, the red regions denote false positive predictions, and the green regions correspond to false negative predictions.

The challenging failure cases are illustrated in Figure 11. For nodules with blurred boundaries, highly irregular morphology and embedding in complex lung textures in the LIDC-IDRI dataset, the model lacks sufficient capability in depicting low-contrast edges, resulting in deviation of partial contours from the ground truth and reduced segmentation integrity. In the FAHGMU-LUNA dataset, when dealing with clinically difficult samples characterized by extremely small targets, low signal-to-noise ratio and being surrounded by dense lung parenchyma textures, the model is prone to slight misjudgment, leading to local under-segmentation or over-segmentation.

The method described in this paper has certain limitations. First, due to the low proportion of lung nodules in CT images, there is a significant imbalance in the distribution of foreground and background samples. This, to some extent, influences the model's capability to precisely characterize small nodules and areas with intricate boundaries. In the future, this class imbalance can be mitigated by introducing more effective data augmentation strategies, thereby improving the model's ability to identify challenging samples and enhancing segmentation robustness. Second, existing methods primarily rely on two-dimensional CT images for segmentation and have not yet fully utilized the inter-slice spatial correlations present in three-dimensional volumetric data. Consequently, there are still limitations in modeling the overall morphology and spatial continuity of lung nodules. Future research will further explore three-dimensional segmentation frameworks to improve the model's capability to represent the 3D structure of lung nodules and their interactions with surrounding tissues. Third, the proposed method employs a 128 × 128 image cropping strategy based on predefined regions, which reduces the input spatial extent to some degree, thereby limiting the model's applicability under scenarios without prior positional information and imposing certain constraints on its deployment in fully real-world clinical workflows. Future work will explore the integration of automatic object localization or candidate region generation modules to enable a unified end-to-end framework from full-image input to segmentation, thereby improving the model's applicability and robustness in real clinical environments.

Despite the aforementioned limitations, DD-Net achieves outstanding segmentation accuracy and stability in lung nodule segmentation tasks, and can provide valuable references for subsequent research on model optimization and computer-aided clinical diagnosis.

## 6 Conclusion

This paper proposes a new lung nodule segmentation framework, DD-Net. Based on a dual-domain encoder-decoder architecture, this method collaboratively performs lung nodule CT image segmentation through multiple modules and introduces a hybrid loss function to enhance the model's optimization capabilities. To enhance the representational capacity of different frequency components and obtain more discriminative frequency-domain features, a frequency-separated module was designed. Additionally, a progressive attention fusion module (PAF) was introduced to integrate multi-layer feature information from both the spatial and frequency domains. Finally, to further strengthen global frequency dependencies and responses to key regions, a frequency self-attention module was proposed. Experiments conducted on public and private clinical datasets indicate that DD-Net performs better than a variety of state-of-the-art methods across multiple evaluation metrics. Ablation experiments further demonstrate the rationality and effectiveness of the module designs, indicating that this method holds great potential for application in the precise screening of early-stage lung cancer.

## Data Availability

The data presented in the study are deposited in the GitHub repository and are publicly available at: https://github.com/1dengli/DD_Net.
